# Graft-versus-tumor effect of post-transplant cyclophosphamide-based allogeneic hematopoietic cell transplantation

**DOI:** 10.3389/fimmu.2024.1403936

**Published:** 2024-06-06

**Authors:** Hirohisa Nakamae

**Affiliations:** Department of Hematology, Osaka Metropolitan University Graduate School of Medicine, Osaka, Japan

**Keywords:** post-transplant cyclophosphamide (PTCy), allogeneic hematopoietic cell transplantation (allo-HCT), HLA-haploidentical transplantation, HLA-matched transplantation, graft-versus-tumor (GVT) effect

## Abstract

Post-transplant cyclophosphamide (PTCy) is becoming the standard prophylaxis for graft-versus-host disease (GVHD) in HLA-haploidentical allogeneic hematopoietic cell transplantation (allo-HCT) and in HLA-matched allo-HCT. Immune reconstitution in the post-transplant setting may influence the graft-versus-tumor (GVT) effect because PTCy has a profound effect on T cell and natural killer cell functions and their reconstitution after allo-HCT. However, many recent studies have shown that the incidence of relapse after allo-HCT with PTCy is comparable to that after conventional allo-HCT. To further improve the outcomes, it is critical to establish a strategy to maintain or effectively induce the GVT effect when using PTCy as a platform for GVHD prophylaxis. However, there is a paucity of studies focusing on the GVT effect in allo-HCT with PTCy. Therefore, focusing on this issue may lead to the establishment of more appropriate strategies to improve transplantation outcomes without exacerbating GVHD, including novel therapies involving cell modification.

## 1 Introduction

Post-transplant cyclophosphamide (PTCy) was developed by researchers at Johns Hopkins University. Since the publication of their results were in 2002, the use of PTCy for graft-versus-host disease (GVHD) prophylaxis has been spreading internationally due to its simplicity and effectiveness (1, 2). Initially, GVHD prophylaxis by PTCy was performed in the context of HLA-haploidentical allogeneic hematopoietic transplantation (allo-HCT), However, since 2004, PTCy has been used at Johns Hopkins University for HLA-matched allo-HCTs. Furthermore, the results of a randomized phase III trial of PTCy in HLA-matched peripheral blood stem cell transplantation (PBSCT) with reduced-intensity conditioning (RIC) (BMT CTN 1703 trial) were recently reported (3). In this trial, GVHD prophylaxis with PTCy was significantly superior to conventional GVHD prophylaxis with tacrolimus (Tac) + methotrexate (MTX) in terms of GVHD-free, relapse-free survival (GRFS). Based on the results of this pivotal trial, PTCy may become one of the new standard prophylaxis for GVHD in HLA -matched PBSCT with RIC.

The mechanism of PTCy-mediated regulation of GVHD was originally proposed based on a major histocompatibility complex (MHC)-matched mouse skin graft model (4). This model involved the selective elimination of activated and proliferating alloreactive T cells by PTCy after transplantation, the clonal disappearance of alloreactive T-cell precursors in the thymus, and the induction of suppressor T cells. However, recent detailed studies on the mechanism of PTCy have revealed that it inhibits the proliferation of alloreactive CD4^+^ effector T cells and impairs the function of residual alloreactive CD4^+^ and CD8^+^ effector T cells, but does not eliminate alloreactive T cells (5). In addition, the thymus was not required for PTCy to exert its effects. Furthermore, PTCy not only effectively prevents acute GVHD by inducing the preferential recovery of regulatory T cells after allo-HCT, but also contributes to long-term tolerance after allo-HCT (5, 6). Aldehyde dehydrogenase isoforms are markedly upregulated in Tregs by up to 10-fold compared to that in conventional CD4^+^ T cells in evaluation systems using the human mixed lymphocyte reaction; Tregs, thus, acquire relative mafosfamide resistance (7). Consequently, Tregs may be more resistant to the *in vivo* active PTCy, mafosfamide than conventional CD4^+^ T cells (8). This may explain the immunological duality of PTCy, wherein cyclophosphamide can act as a pro- and anti-inflammatory agent, depending on the circumstances (9). In addition, aldehyde dehydrogenase-1A1 expression in CD8+ T cells is increased in allogeneic reactions in patients; CD8+ T also rely in part on aldehyde dehydrogenase for protection against PTCy, and their recovery is more robust than conventional CD4+ T cells. They can therefore help prevent relapses after allo-HCT (10). In contrast, Ritacco et al. confirmed *in vitro* that activated/proliferating Tregs were highly sensitive to mafosfamide, and that PTCy depleted KI67^+^ Tregs in the humanized mouse model (11). They also demonstrated that even though proliferating Tregs were depleted by PTCy, a higher proportion of Tregs was enriched because mice receiving PTCy had higher levels of IL-2 and, in contrast, lower levels of IFN-γ and TNF-α. In addition, they showed that Tregs were not needed for GVHD prevention by PTCy and although PTCy decreased the graft-versus leukemia effects, it did not abolish it.

Nevertheless, it was reported that PTCy impaired immune reconstitution and dysfunction of T cells and natural killer (NK) cells, especially in the early phase of transplantation, and thereby led to an increase in the incidence of infections after allo-HCT, such as those involving CMV and other viruses (12, 13). Thus it cannot be completely ruled out that PTCy hinders T-cell functions involved in the graft-versus-tumor (GVT) effect.

Patients who developed maximum-severity grade II aGVHD, compared with those who did not, were shown to have a significantly lower relapse rate following HLA-matched allo-HCT using PTCy, suggesting that both GVHD-dependent and -independent GVT effects may exist in PTCy based allo-HCT (14, 15) and it is possible that PTCy cannot effectively separate harmful alloreactive reactions and the beneficial GVT effect since GVHD can contribute to the GVT effect. By contrast, an important finding is that a large population analysis of the European Society for Blood and Marrow Transplantation (EBMT) registry data of patients with acute myeloid leukemia (AML) undergoing PTCy-haplo did not show that grade II acute GVHD was associated with relapse incidence (16); therefore, it was not determined whether the development of GVHD contributed to the GVT effect in PTCy-based allo-HCT.

A recent meta-analysis of over 20,000 patients showed that HLA-haploidentical allo-HCT using PTCy (PTCy-haplo) was associated with a slightly higher risk of relapse compared with HLA-matched unrelated allo-HCT (17). An analysis of Japanese registry data also indicated a higher relapse rate with PTCy-haplo than with unrelated transplants (18). Inoue et al. showed that in patients with AML that was not in morphological remission, those receiving PTCy-haplo had a higher relapse rate than those receiving HLA-haploidentical allo-HCT with peri-transplant glucocorticoid administration (19). Nevertheless, allo-HCT using PTCy results in good control of both acute and chronic GVHD and leads to a low incidence of non-relapse mortality (NRM).

Against this background, a question for the future is how to effectively induce the GVT effect in the context of PTCy-based GVHD prophylaxis. By determining optimal allo-HCT settings, it may be possible to create environments that maximize the GVT effect when using PTCy and establish an effective algorithm for donor selection.

A common argument is that the adverse prognostic impact of GVHD and relapse rate (which is affected by the GVT effect) are simultaneously assessed as GRFS. However, it is important to have discussions that focus on the GVT effect, and debate from this perspective is very limited. With the aim of establishing effective strategies to reduce relapse/progression in PTCy-based allo-HCT, this review evaluates the GVT effect of allo-HCT using PTCy, as well as the impact of PTCy on relapse after allo-HCT, from various perspectives using data of numerous studies.

## 2 The potential effect of PTCy on effector cells involved in the GVT effect

As mentioned above, it was initially proposed that PTCy acts by selectively eliminating harmful alloreactive T cells, primarily by their direct destruction and secondarily by intrathymic clonal deletion of their precursors (4). According to these mechanisms, the GVT effect can persist after PTCy administration because tumor-specific T cells may be less affected than alloreactive T cells, which respond more intensely to ubiquitous alloantigens.

However, the newly proposed theory regarding the effect of PTCy is that the drug does not eliminate alloreative T-cells, and that it can negatively affect the function of both non-alloreactive and alloreative T cells to some degree (5, 20). The extent of the influence of PTCy on the GVT effect remains unclear.

We previously demonstrated that NK cell counts on days 30 and 60 were lower in PTCy-haplo than in HLA-matched related allo-HCT, and the ratio of regulatory T cells (Tregs) to conventional CD4^+^ T cells was higher in PTCy-haplo up to and including day 90 (6). Rambaldi et al. also demonstrated that the number of NK cells decreased early after PTCy-haplo, with preferential expansion of immature CD56^bright^CD16^-^ NK cells (13). Additionally, early T-cell recovery was delayed in PTCy-haplo, with a marked reduction in naive T cells, and early after PTCy-haplo there was a higher ratio of Tregs to conventional T cells as well as increased PD-1 expression on memory T cells. An increased ratio of Tregs to conventional T cells and increased PD-1 expression may contribute to the induction of immune tolerance, whereas an unbalanced immune recovery of T and NK cells may attenuate the GVT effect.

Zhao et al. showed that the expression of inhibitory receptors, including TIM-3, CD38, and CD39, was markedly upregulated on both CD4^+^ and CD8^+^ T cells on day 30 in patients receiving PTCy (21). Upregulation of PD-1 on CD8^+^ T cells with compromised function was maintained until day 180. Importantly, the authors also noted that the expression of granzyme B and perforin on CD8^+^ T cells was significantly lower in patients with disease relapse.

Moreover, in MHC mismatch murine transplantation experiments, we demonstrated that after allo-HCT, PTCy compromised GVT efficacy and ameliorated GVHD by suppressing donor CD8^+^/CD4^+^ alloreactive T cells expressing PD-1 (22). Nakamura et al. also demonstrated that in recipient mice with a high tumor burden, a PTCy dose of 50 mg/kg may decrease the GVT effect, leading to tumor-related death (23).

The results of these studies raise concerns about the impact on GVT effect, however, relapse rates are comparable between PTCy-haplo and HLA-matched allo-HCT (24–28).

Additionally, a recent randomized phase III trial of PTCy in patients with HLA-matched PBSCT (BMT CTN 1703 trial) did not show an increased incidence of relapse in the PTCy arm (3). The HOVON-96 trial also revealed no impact of PTCy on relapse: the cumulative incidence of relapse at 3 years post-transplant was 24% in the cyclosporine A (CyA)/mycophenolic acid group and 24% in the PTCy/CyA group (29). Furthermore, Brissot et al. recently reported the results of a randomized, open-label, multicenter, phase II trial of allo-HCT from a PBSC graft from an HLA-matched sibling or 10/10 HLA-matched unrelated donor using RIC. The cumulative incidence of relapse was similar in the PTCy and ATG groups (30).

These results suggest that in cases of remission with or without measurable or minimal residual tumor or indolent progressive disease, the GVT effect may not be impaired by PTCy to a clinically relevant extent, although at this point, the extent to which PTCy impacts the GVT effect remains unclear.

## 3 Assessment of the association between donor/graft characteristics and the GVT effect in PTCy-based allo-HCT

The incidence of relapse or progression after allo-HCT has been used as a surrogate indicator of the GVT effect. This incidence can be affected not only donor/graft characteristics and the types of immunosuppressive drugs, including PTCy, but also other factors such as conditioning intensity, disease risk, and tumor burden at transplantation. Therefore, evaluating the strength of the GVT effect exerted specifically by grafts in the PTCy-based setting necessitates reviewing the results of a randomized controlled trial or matched-pair analysis, or after adjustment for other factors that may affect relapse as in a multivariate analysis.

### 3.1 The impact of donor age on relapse

Many studies and guidelines state that transplantation from younger donors is associated with a reduced incidence of acute or chronic GVHD and better OS (31–33). However, the effect of donor age on relapse has not been determined.

Among non-HLA donor characteristics, a relationship between donor age and relapse has frequently been reported in PTCy-haplo, although the results have been controversial (34–44). Unexpectedly, a few papers have shown that the relapse rate decreases with increasing donor age (34–36). It is still uncertain whether younger donors should be prioritized in PTCy-based allo-HCT.

Canaani et al. performed a retrospective analysis in a cohort of 1,270 patients with AML or acute lymphoblastic leukemia (ALL) who underwent HLA-haploidentical allo-HCT with PTCy or other GVHD prophylaxis, and showed that among patients younger than 40 years, donor age above 55 years was independently associated with a nonsignificantly higher risk of relapse (hazard ratio [HR],1.85, P=.058) (37). Ciurea et al. also reported that among patients with AML or myelodysplastic syndrome (MDS) over age 55 years, receiving PTCy-haplo, progression-free survival (PFS) was most favorable in patients with intermediate- or good-risk cytogenetics, in first or second remission, who underwent allo-HCT from a younger donor ≤ 40 years (HR, 0.2, P = .01) (38).

In recent reports from the Acute Leukemia Working Party of EBMT, Sanz et al. showed that although no specific variable was significantly related to the risk of relapse in AML patients receiving PTCy-haplo, older and female donors for male recipients showed a significantly negative impact on GRFS, and that younger and male donors for male recipients should be preferred when possible (39). In addition, they showed that in PTCy-based allo-HCT from 10/10 HLA-matched and 9/10 HLA-mismatched unrelated donors in AML patients in first or second complete remission, donor age more than 30 years had a significantly adverse effect on risk of relapse (HR 1.38; 95%Cl 1.06-1.8) (40).

On the other hand, in a European multicenter retrospective analysis of 990 patients who underwent PTCy-haplo, Marioti et al. showed that while older donor age was nonsignificantly related to increased NRM (HR, 1.05, P=.057), it was associated with a significantly decreased risk of disease relapse (HR, 0.92, P=.001) (34). In a multivariate analysis of patients with acute lymphoblastic leukemia (ALL), Mehta et al. noted that haploidentical HCT in younger donors was related to a higher risk of relapse (35). Pruitt et al. reported that rising donor age was associated with lower risk of relapse but greater NRM in 299 patients undergoing peripheral blood PTCy haplo-PBSCT (36).

However, the possibility of decreased risk of relapse with increasing donor age, if true, might be explainable by an increase in NRM as a competing risk of relapse.

Other studies found no relationship between donor age and incidence of relapse (41–44). Despite the conflicting results, however, it is possible that an optimal donor age may exist in PTCy-haplo.

### 3.2 The impact of the recipient–donor relationship on relapse

There have been conflicting results regarding how the recipient–donor relationship affects the incidence of relapse in PTCy-haplo. Solomon et al. reported that this relationship was significantly related to risk of relapse (child vs. parent, HR, 0.27, P=.009; sibling vs. parent, HR, 0.38, P=.035) (45). de Lima et al. showed that when the mother was the donor, the relapse rate was higher than with other donors (HR, 2.52, P=.05) (46). In contrast, DeZern et al. and Danylesko et al. demonstrated that donor relationship was not associated with risk of relapse (41, 47).

In HLA-haploidentical allo-HCT, non-first-degree donors should be considered as alternatives to first-degree donors. A report from Johns Hopkins Hospital showed that PTCy-haplo patients with nonmyeloablative BMT from non-first-degree relatives (including second- or third-degree related donors) had a comparable prognosis after transplantation to that with a first-degree relative as a donor, including the relapse incidence and incidence of GVHD and NRM (48). The analysis of patients with AML or ALL who achieved complete remission after receiving the first HLA-haploidentical allo-HCT (including PTCy-haplo) using the EBMT registry data did not show significant differences in prognosis, including relapse incidence and GRFS following HLA-haploidentical allo-HCT from non-first-degree and first-degree related donor (49).

These results indicate that a non-first-degree haploidentical donor may be a potential alternative when a first-degree haploidentical donor is unavailable; however, there are no data showing that allo-HCT from a non-first-degree donor leads to an enhanced GVT effect.

As data accumulate in the future, it may be possible to establish an algorithm to select the best donors for PTCy-haplo.

## 4 The effect on relapse by donor source: bone marrow versus peripheral blood stem cells

PB stem cell grafts contain larger numbers of myeloid precursors and lymphocytes than bone marrow (BM) grafts. In addition, it was reported that BM grafts contained a higher percentage of Tregs than PB (50). Therefore, the GVT effect mediated by donor cells in PB grafts may be greater than that derived from donor cells in BM.

O’Donnell et al. performed a matched-pair analysis and showed that in PTCy-haplo with non-myeloablative conditioning, PB was significantly associated with a lower risk of relapse than BM at 1 year (19% vs 49%, respectively, P=.002), 2 years (24% vs 51%, respectively, P=.006), and 3 years (24% vs 58%, respectively, P<.001) (51). Bashey et al. demonstrated that PTCy-haplo with BM showed a higher risk of relapse in patients with leukemia compared to that with PB (HR, 1.73, P=.002) but not lymphoma (HR, 0.87, P=.64) (52). Sharma et al. also reported in PTCy-haplo, BM was associated with a significantly higher risk of relapse at 2 years compared with PB (36% vs. 16%, respectively, P=.03) (53). Furthermore, a recent large meta-analysis comparing BM with PB in PTCy-haplo showed that the risk of relapse was 16% lower in the PB graft group, indicating a significant difference (HR, 0.84, P=.001) (54).

Conversely, many other investigators reported that PB and BM grafts did not result in different incidences of relapse in PTCy-haplo (55–61). The reasons for these conflicting results are unclear, but may include differences in transplantation procedures, patient and disease characteristics, sample power, and infused cell doses. Additionally, NRM tends to be more common in PB than in BM grafts (59), making it debatable whether PB should be prioritized for relapse control.

## 5 The impact of donor–recipient HLA disparity on relapse

It has been reported that donor-recipient HLA-B leader match, HLA-DRB1 and HLA-DQB1 GVH direction match, and HLA-DPB1 T-cell epitope (TCE) match status can affect the prognosis of PTCy-haplo (62–64). On the basis on these analyses, a tool to predict disease-free survival after PTCy-haplo for acute leukemia and MDS has been published by the Center for International Blood and Marrow Transplant Research (CIBMTR) (URL: https://haplodonorselector.b12x.org/v1.0/). Herein, among the HLA-related factors, those reported to be relevant to relapse are discussed.

### 5.1 HLA-DRB1 and HLA-DPB1

The Johns Hopkins University group reported that in PTCy-haplo bone marrow transplantation (BMT) with non-myeloablative conditioning, each increment of HLA mismatch between donor and recipient reduced the risk of death or relapse by 20% (65). In addition, one HLA-DRB1 antigen mismatch in the GVH direction and two or more HLA class I mismatches, including HLA-A, -B, and –Cw, in either the GVH or host-versus-graft (HVG) direction, were related to a lower frequency of relapse.

Solomon et al. demonstrated that a higher number of HLA mismatches (x/10) in the GVH direction (HR, 0.29, P=.001, 4/5 vs. ≤ 3), and the presence of an HLA-DPB1 nonpermissive mismatch (HR, 0.29, P=.001), were both associated with reduced risk of relapse/progression in PTCy-haplo (45). In a multivariate analysis, this group also showed that a HLA mismatch at HLA-DR in the GVH direction, and a nonpermissive HLA-DP mismatch, were both independently and significantly related to a lower risk of relapse (63).

A group at the University of Texas MD Anderson Cancer Center sought to determine the clinical impact of HLA-DPB1 molecular mismatch as quantified by mismatched epitopes and predicted indirect HLA epitope recognition scores (64). They analyzed a cohort of 1,514 patients, including 12.6% who received PTCy-based transplantation, who received allo-HCT from unrelated donors matched at HLA-A, -B, -C, -DRB1/3/4/5, and -DQB1 loci. They demonstrated a tendency toward a lower risk of relapse associated with HLA-DPB1 mismatched eplets in the GVH direction.

All of the aforementioned studies were performed at a single center. The results of multivariable analysis in a large cohort study using a CIBMTR database of 1,434 acute leukemia or MDS patients receiving PTCy-haplo showed a significant association between HLA-DRB1 mismatches in GVH direction and a lower risk of disease relapse (HR, 0.65, P<.0001) (62).

### 5.2 HLA-B leader

The transplant recipient’s HLA-B leader genotype is either methionine (M)/M, M/threonine (T), or T/T. HLA-E activates NK cells via the inhibitory receptor NKG2A, and the strength of signaling along the NKG2A–HLA-E axis depends on the dimorphism at position -21 of the HLA-B leader sequence, with -21M promoting higher HLA-E expression than -21T (66–68). NK cells educated with the M+ HLA-B leader genotype are thought to be more cytotoxic, more mature/activated, and to secrete more cytokines when exposed to target cells when compared with NK cells educated with the T/T genotype (69, 70). HLA-E expression activates CD8+ T cells via T-cell receptors, and T cells (together with NK cells) may contribute to HLA-B leader-mediated immune responses (71). Solomon et al. reported that in an analysis of the relationship between the HLA-B leader genotype and relapse, the presence of an HLA B-leader containing M at position -21 of the leader sequence in the recipient (vs. M-) reduced the cumulative incidence of relapse (16% vs. 42%, respectively), and this effect was found primarily in lymphoid malignancies (70). However, an analysis based on PTCy-haplo registry data of the CIBMTR showed that HLA-B leader match, in comparison with the mismatch, was significantly associated with superior OS and lower transplant-related mortality but not relapse incidence (62).

Furthermore, our recent single-center analysis of PTCy-haplo showed that the impact of the recipient HLA-B leader genotype was altered by the PTCy dose: recipients with the M+ leader showed a decreased incidence of relapse in the high-dose group (total PTCy dose of 75–100 mg/kg) but not in the low-dose group (less than 75 mg/kg PTCy) (72).

### 5.3 HLA T-cell epitope

HLA-DPB1 mismatches have been classified as permissive or non-permissive using a functional toxicity assay and TCE similarity analysis (73, 74). Zou J et al. analyzed a cohort of 1,514 patients who received HLA-matched unrelated allo-HCT, including small cohort who received PTCy, and showed that in the permissive mismatch subgroup categorized by TCE, high HVG mismatched eplet and the Predicted Indirectly Recognizable HLA Epitopes Score was correlated with an increased risk of relapse (HR, 1.36, P=.026 for ME) (64).

In contrast, the CIBMTR registry data for PTCy-haplo showed that HLA-DPB1 TCE nonpermissive mismatch was not significantly associated with the incidence of relapse when compared with HLA-DPB1 TCE match or TCE permissive mismatch (62).

## 6 The effect of alloreactivity of NK cells on the GVT effect in PTCy-haplo

The NK cell–mediated GVT effect is thought to be exerted without aggravating GVHD. A report from a group in France of 144 patients who underwent PTCy-haplo BMT or PBSCT demonstrated a significantly reduced risk of relapse (HR, 0.21, P=.013) and improved PFS in the group with hematological disease in non-remission who underwent PTCy-haplo with killer cell immunoglobulin-like receptor (KIR) ligand–incompatible donor-recipient pairs (HR, 0.42, P=.028) (75). In contrast, this effect of KIR ligand incompatibility was not observed in the group with hematological disease in the remission. However, a multicenter retrospective analysis by EBMT demonstrated that in the PTCy setting, KIR ligand mismatching was associated with worse survival (HR, 1.41, P=.03) and a trend toward higher relapse (HR, 1.36, P=.09) when comparing with KIR matching. the results in these reports were based on the classical KIR ligand incompatibility model (76).

Investigators at Nantes University Hospital in France performed a retrospective study of the impact of KIR/HLA incompatibilities on clinical outcomes in PTCy haplo-PBSCT with RIC (77). They used the receptor-ligand model and showed that KIR2DL/HLA incompatibilities, compared with the lack of such incompatibilities, were significantly associated with a higher incidence of acute GVHD (72.7% vs. 45.7%, respectively, P=.04) and lower incidence of relapse (6.2% vs. 42.8%, respectively, P=.008).

Importantly, the GVHD prophylaxis strategy may affect the GVT effect exerted through NK cell alloreactivity. For instance, a Kanazawa University group examined the effects of CyA, Tac, mycophenolic acid [an active form of mycophenolate mofetil (MMF)], and MTX on the proliferation and cytotoxicity of NK cells (78). They demonstrated that under incubation of NK cells and IL-2, mycophenolic acid could inhibit the function of NK cells by decreasing the proportion of CD16^-^CD56^bright^ cells, thus reducing the expression of activated NK cell receptors and suppressing the downregulation of p27 on NK cells. MMF has often been used in triplet GVHD prophylaxis in PTCy-haplo, and it may reduce the GVT effect through NK cell alloreactivity.

Russo et al. reported that NK cell alloreactivity did not contribute to prognosis because PTCy eliminates mature donor NK cells that are partially responsible for this alloreactivity (79). The relapse rate was significantly lower in patients with higher killer cell immunoglobulin-like receptor (KIR) positivity rates in the remaining mature NK cells at 30 days post-transplant, suggesting that PTCy may attenuate the GVT effect of mature NK cells. Furthermore, an investigation by Willem et al. in 51 patients who underwent PTCy-haplo using PB demonstrated that genetic KIR2DL/HLA incompatibilities were significantly associated with reduced relapse, but recipients with inhibitory KIR/HLA mismatches exhibited a significantly decreased count of KIR2DL2/3^+^NK cells at day 30, indicating that reactive KIR NK cells are specifically targeted by PTCy (77).

The combination of KIR genotyping and HLA typing could result in a more accurate estimate of the possible benefits of NK alloreactivity through NK cell licensing than a model that considers the only presence of KIR and KIR ligands (80). An MD Anderson group applied the count functional inhibitory KIR (CF-iKIR) score, an additive model that incorporates multiple inhibitory KIRs and their corresponding KIR ligands. This group analyzed 354 patients with hematologic malignancies who received PTCy-haplo, and found that a higher CF-iKIR score was associated with improved prognosis due to the combined effects of both relapse prevention and NRM.

In 2010, a Johns Hopkins University group reported that patients homozygous for the KIR A haplotype who received PTCy-haplo from a donor who had at least one KIR B haplotype had significantly better OS (HR, 0.30, P=.01), event-free survival (HR, 0.47, 95% CI 0.22-1.00), and NRM (cause-specific HR, 0.13, P=.046) than those without KIR B haplotype (81).

KIR2DS1 is an activating KIR. At our institution we performed genotype analysis of 16 KIR genes in 91 patients with hematopoietic malignancies in complete remission who underwent PBSCT from an HLA-haploidentical donor with reduced-dose PTCy (82). Patients who received PTCy-haplo from a KIR2DS1-positive donor, compared to those with a KIR2DS1-negative donor, had significantly lower relapse incidences at 1 and 2 years (0% vs. 32.6%, respectively, and 9.2% vs. 42%, respectively) and had significantly better 1- and 2-year OS (91.7% vs. 58.7%, respectively, and 83% vs. 34%, respectively). However, this effect was not observed in patients who received PTCy-haplo while not in remission. Our results imply that the GVT effect in PTCy-haplo was exerted via donor KIR2DS1 in patients with a low tumor burden.

It is important to note that the benefit of NK cell alloreactivity is more potent in AML than in acute lymphoblastic leukemia (83). NK alloreactivity has shown broad efficacy in PTCy-haplo across disease types other than AML, although the reason is unknown, suggesting the complexity of analyzing the alloreactivity of NK cells via inhibitory KIRs.

In addition, NKG2D is expressed on various immune cells, including NK cells, where it functions as an activating receptor. In our study, PTCy-haplo from donors with NKG2D rs1049174 CC was associated with a significantly decreased risk of relapse/progression (adjusted HR, 0.2, P=.007) when compared those with NKG2D rs1049174 GG. A positive effect of the rs1049174 CC donor on relapse/progression was noted in patients with diseases other than AML (84).

## 7 The influence of infused cell doses and/or composition on relapse in PTCy-based allo-HCT

In a prospective study of HLA-haploidentical PBSCT with modified doses of PTCy, we recently reported that the number of infused CD34^+^ and CD3^+^ cells affected the incidence of relapse in a nonlinear fashion, and that there might be optimal numbers of infused CD34^+^ and CD3^+^ cells (85). Importantly, that study involved a non-linear restricted cubic spline Cox regression analysis, and showed that a graft composition of > 4.54 × 10^6^/kg CD34^+^ cells and > 1.85 × 10^8^/kg but ≤ 3.70 × 10^8^/kg CD3^+^ cells was significantly associated with a lower incidence of relapse/progression (HR, 0.31, P=.038).

The EBMT performed a retrospective study of T cell–replete haploidentical PBSCT, including PTCy, for acute myeloid leukemia, and demonstrated that a high CD34^+^ cell dose of > 4.96 × 10^6^/kg was significantly associated with lower NRM and better overall and leukemia-free survival, but not with relapse incidence (86). Elmariah et al. performed a retrospective study of PTCy-based PBSCT and showed that infused CD34^+^ cell doses > 5 × 10^6^/kg resulted in lower NRM and improved OS and PFS, but there was no association with relapse incidence (87). Furthermore, several retrospective studies have shown that the number of infused CD34^+^ cells or CD3^+^ cells had no significant impact on survival or relapse (15, 88–91).

On the other hand, one study performed cluster discrimination and found that patients receiving grafts containing high levels of CD3^+^ and NK cells showed significantly worse event-free survival than low levels of CD3^+^ and NK cells (85% vs. 61%, P=.0393) due to a higher incidence of relapse (36% vs.12%, P=.0105) in HLA-matched allo-HCT wth PTCy (92).

We speculate that these contradictory results might be caused by the heterogeneity of PTCy-based transplantation procedures, including HLA-matched vs. HLA-mismatched, BMT vs. PBSCT, different disease statuses, and different study settings (e.g., prospective vs. retrospective). However, we think that the most important explanation is the variety of analytical approaches used. Most previous studies applied linear models and/or performed analyses based on the number of infused cells, but we suppose that the numbers of infused CD34^+^ and CD3^+^ cells affect outcomes in a nonlinear fashion.

## 8 The possible impact of PTCy dose on the GVT effect

There are several reasons for selecting the standard dose and timing of PTCy administration. First, in an MHC-matched mouse skin transplant model, a high dose of cyclophosphamide was required, and in humans 50 mg/kg is almost the maximum tolerated high-dose therapy for aplastic anemia. Second, in terms of preventing graft rejection in the mouse skin transplant model, the dose effect is equivalent on days 2 and 3, and day 3 was selected to maximize the interval between conditioning and PTCy and to minimize toxicity. Third, a phase I/II study of PTCy-haplo with BM using non-myeloablative conditioning suggested a lower risk of extensive chronic GVHD in the two-dose PTCy group when 50 mg/kg was given on day 3 and 50 mg/kg on days 3 and 4 (93, 94). Thus, the optimal dose of PTCy has not been adequately evaluated clinically, and there is an emerging argument that the standard PTCy dose may be too high and therefore cause increased toxicity, delayed graft engraftment, and impaired immune reconstitution after allo-HCT.

In this context, it is important to note that a comparative analysis of standard-dose PTCy (total 100 mg/kg) and reduced-dose PTCy (total 80 mg/kg) in PTCy-haplo, in a study based on Japanese registry data, showed no significant difference in OS or the incidence of acute or chronic GVHD, and the major outcomes were similar (95). Therefore the benefits of reducing the PTCy dose to 80 mg/kg are unclear. On the other hand, a single-center study from France compared the results of 80 mg/kg of PTCy versus 100 mg/kg of PTCy in elderly patients 65 years and older or those 60 years and older with a history of cardiac events, and multivariate analysis showed that despite the small number of patients, the 80 mg/kg PTCy dose was associated with significant improvement in neutrophil and platelet engraftment and a trend toward a lower incidence of BK virus-associated hemorrhagic cystitis (96). Of note, the two aforementioned studies showed that reducing the PTCy dose to 80 mg/kg had no impact of relapse.

We have long suspected that PTCy may attenuate the GVT effect, and have used a reduced dose of PTCy in prospective trials (85, 97). Our institution was the first in Japan to introduce PTCy-haplo, and we have used a reduced dose of PTCy since 2009 with the aim of enhancing the GVT effect and reducing PTCy toxicity. Based on our study results, we assume that a dose reduction to 75 mg/kg (50 mg/kg on day 3 and 25 mg/kg on day 4) is possible even when PB is used as a stem cell source (85). However, there is no clear evidence that reducing PTCy dose enhances the GVT effect.

Recently, Kanakry et al. showed that the optimal dose of PTCy was 25 mg/kg on days 3 and 4 post-transplant in murine experiments (5). They also showed that that PTCy exhibited maximum efficacy when administered on day +4, indicating that PTCy administered on day +4 alone may be as effective as PTCy administered on days +3 and +4 at the optimized dose (94).

It is important to note that the optimal dose of PTCy may vary depending on whether the stem cell source is BM or PB. A phase I/II trial on PTCy haplo BMT was performed by the National Institutes of Health, and the results were reported at the American Society of Hematology meeting in 2021 (98). In this trial, a 3 + 3 dose escalation design was introduced for PTCy administered at three doses: 50 mg/kg on days 3 and 4 (dose level 1: DL1), 25 mg/kg on days 3 and 4 (DL2), and 25 mg/kg on day 4 only (DL3). The median times to neutrophil and platelet engraftment were significantly faster in DL2 compared with DL1, while control of severe acute GVHD was possible with DL2 and DL3. In addition, patients receiving low-dose PTCy, DL2 or DL3 demonstrated low severity and duration of mucosal damage, low cytomegalovirus reactivation rate, and short duration of symptomatic BK virus-associated cystitis, suggesting that PTCy-haplo BMT may allow for further reduction of PTCy.

Currently, the standard dose of PTCy is 50 mg/kg on days 3 and 4 after transplantation, but various doses of PTCy have been used, particularly to reduce toxicity. However, it is uncertain whether the reducing the PTCy dose is associated with improvement in the GVT effect. Ongoing clinical trials involving PTCy dose modification are summarized in **Table 1**.

**Table 1:** Clinical trials on dose modification of post-transplant cyclophosphamide.

Trial ID	Phase	Title of trial	Intervention	Locations
NCT06000982	P3	Comparison of Different Dose of Post-transplantation Cyclophosphamide as Graft Versus Host Disease Prophylaxis	PTCy 40mg/kg vs. PTCy 50mg/kg	Shenzhen People’s Hospital, China Rui Jin Hospital, China
NCT03395860	P2	Low Dose ATG Plus Low Dose PTCy as GVHD Prophylaxis in Haplo-HSCT	ATG 5 mg/kg and one dose of PTCy 50mg/kg	Shanghai First People’s Hospital Shanghai, China
NCT03983850	P1, 2	Optimizing PTCy Dose and Timing	PTCy at 25 mg/kg/day on days +3 and +4, and PTCy 25 mg/kg on day +4 to assess for safety and determine Phase II dose	NIH Clinical Center, Bethesda, Maryland, United States
NCT06108739	Randomized, P3	ATG Plus Low-dose PT-Cy for GVHD Prevention	ATG 10mg/kg and two doses of PTCy 14.5 mg/kg days 3 and 4 in ATG-PTCy cohort.	Peking University People’s Hospital, China
NCT05622318	P2	De-escalated Cyclophosphamide (PTCy) and Ruxolitinib for Graft-versus-Host Disease (GVHD) Prophylaxis	PTCy at 25 mg/kg on Day +3 and +4, and ruxolitinib 5 mg (IV) twice daily starting after engraftment	Froedtert Hospital & the Medical College of Wisconsin, United States
NCT05158608	Randomized, P3	Comparison of PT-Cy at a Dose of 25 mg/kg/Day and PT-Cy at a Dose of 50 mg/kg/Day in GVHD Prophylaxis	PTCy 25 mg/kg/day vs. PTCy 50 mg/kg/day	National Research Center for Hematology, Russia
NCT05436418	P1, 2	The Lowest Effective Dose of Post-Transplantation Cyclophosphamide in Combination With Sirolimus and Mycophenolate Mofetil as Graft-Versu0s-Host Disease Prophylaxis After Reduced Intensity Conditioning and Peripheral Blood Stem Cell Transplantation	PTCy based on dose level being tested (50, 35, 25, or 15 mg/kg) IV once daily over 2 hours on days +3 and +4.	City of Hope, United States NIH Clinical Center, United States Fred Hutch Cancer Center, United States
NCT04959175	P1, 2	Phase I/II Study to Reduce Post-transplantation Cyclophosphamide Dosing for Older or Unfit Patients Undergoing Bone Marrow Transplantation for Hematologic Malignancies	PTCy 25 mg/kg/day on days +3 and +4.	NIH Clinical Center, United States Hospital of the University of Pennsylvania, United States
NCT05780554	NA	Post-transplantation Cyclophosphamide in Haploidentical Stem Cell Allografts Dose Reduction: 50 mg/kg vs 25 mg/kg	PTCy 25 mg/kg/day or 50 mg/kg/day on days +3 and +4.	Centro de Hematología y Medicina Interna, Mexico
NCT06041893	NA	Haploidentical Hematopoietic Stem Cell Transplantation With Early ATG and Low Dose Post-transplant Cyclophosphamide	ATG, low dose PTCy	Samsung Medical Center, Korea

PTCy, post-transplant cyclophosphamide; ATG, anti-T-lymphocyte globulin; iv, intravenous injection.

Interestingly, an interim report of Phase I/II of intermediate-dose PTCy after RIC (NCT03983850) was presented by McCurdy et al. (99, 100). The results showed that an intermediate-dose PTCy was associated with a lower incidence of GVHD and faster engraftment than standard-dose PTCy even in a cohort of high-risk patients, 25% of whom were aged 75 years or older. The relapse rate was low, presumably owing to the lower doses of PTCy or shorter duration of sirolimus after allo-HCT. They also reported that an intermediate-dose PTCy was associated with low toxicity and allowed for early discontinuation of immunosuppression in an older and unfit cohort.

## 9 Do the drugs administered in combination with PTCy impact the GVT effect?

In PTCy-haplo, the standard triple drug combination of PTCy, tacrolimus, and MMF has been widely used. However, it is uncertain whether HLA-matched allo-HCT with PTCy requires a similar intensity of triple-drug GVHD prophylaxis as PTCy-haplo.

The EBMT group analyzed 423 acute leukemia patients who underwent allo-HCT from HLA-matched sibling donor (MSD) and unrelated donors who received PTCy alone or in combination with other immunosuppressive agents for GVHD prophylaxis, and divided these patients into three groups: PTCy alone (group 1), PTCy plus one of CyA/MTX/MMF (group 2), and PTCy plus either CyA + MTX or CyA + MMF (group 3) (101). In multivariate analysis, compared to PTCy alone, PTCy plus two immunosuppressive agents was significantly associated with a lower risk of extensive chronic GVHD and lower NRM, and improved OS. However, the number of immunosuppressive agents added to PTCy had no effect on the incidence of relapse.

In a recent pivotal, multicenter, randomized trial in HLA-matched allo-HCT (BMT CTN 1703 trial), PTCy + Tac + MMF triple-drug GVHD prophylaxis was not associated with an increased incidence of relapse compared with conventional prophylaxis with Tac + MTX (3).

On the other hand, Mehta et al. compared a group treated with PTCy + Tac with a group that received PTCy + Tac + MMF in the context of HLA-matched allo-HCT (102). The three drug group did not exhibit a lower relapse rate than the two-drug group, but did demonstrate a significantly greater risk of grade II-IV acute GVHD, as well as prolonged neutrophil engraftment by 2 days and an increased risk of bacterial infection. There are no previous reports showing that the addition of MMF for GVHD prevention was associated with a significantly worse GVT response. However, the HOVON-96 trial showed that a single agent of CyA in addition to PTCy without MMF could regulate GVHD in HLA-matched allo-HCT with PTCy (29).

The combination of low-dose PTCy and ATG has a marked effect on Tregs (103), and combination therapy has been used in the hope of synergistic effects of the combination of PTCy and ATG (104). Furthermore, the combination of PTCy and ATG is expected to suppress relapse owing to the rapid reconstitution of some NK cell subsets. However, there are no reports of a significant reduction in relapse incidence with the combination of PTCy and ATG, although there are reports of improvements in GRFS (105).

Further investigation is needed to determine whether the use of strong immunosuppressive agents for patients in non-remission or re-transplanted patients undergoing HLA-matched allo-HCT with PTCy has a negative impact on disease progression post allo-HCT.

## 10 Early initiation of calcineurin inhibitor in PTCy framework in HLA-haploidentical transplantation

The Johns Hopkins University group that developed PTCy-haplo initially used non-myeloablative conditioning with bone marrow as the stem cell source (93). PTCy was administered at 50 m/kg on days 3 and 4 post allo-HCT, while Tac and MMF were administered starting on day 5 post allo-HCT. However, the relapse rate of 46% at 2 years was very high. A Genova, Italy group was the first to integrate myeloablative conditioning into the PTCy-haplo regimen for disease control. They introduced 50 mg/kg of PTCy on days 3 and 5 post-transplant with a 1-day rest day (106), and started CyA on day -1 or day 0, with initiation of MMF on day 1 after allo-HCT.

A retrospective study of the prognostic impact of different PTCy schedules, immunosuppressant combinations, and timings was performed by the EBMT (107). This study compared three groups, as follows: group 1, PTCy on days +3 and +4, followed by tacrolimus + MMF starting on day +5; group 2, PTCy on days +3 and +4, followed by CyA + MMF starting on day +5; and group 3, PTCy on days +3 and +5 with early initiation of CyA on either day -1 or 0, followed by MMF on day 1. Group 3 showed significantly higher leukemia-free survival and a lower incidence of relapse than groups 1. The study showed that while treatment with the Genova approach was associated with an increased incidence of acute and chronic GVHD compared to the standard PTCy haplo approach in patients with AML, it correlated with a significantly lower percentage of patients who died with relapse (44% and 23%, respectively) (107, 108).

In Japan we conducted a multicenter, prospective study of PTCy-haplo for adults with T-cell leukemia/lymphoma (ATLL), in which we performed early initiation of immunosuppressive agents, as in the Genova approach (109). We showed relatively favorable outcomes, with a disease progression rate of 28% at 1 year and a 2-year OS rate of 73%, despite the fact that ATLL is a highly aggressive malignant lymphoma. In the future, prospective trials on the timing of immunosuppressive drug initiation in PTCy-based allo-HCT are needed to draw definitive conclusions regarding the optimal point at which to start treatment with immunosuppressive agents.

## 11 Differences in immune reconstitution between PTCy-haplo and HLA-matched allo-HCT using PTCy and their impact on GVT effect

As PTCy is now being applied to HLA-matched allo-HCT, it will be clinically important to compare PTCy-haplo with HLA-matched allo-HCT using PTCy to determine whether the effect of PTCy on GVT varies with the degree of HLA disparity between the recipient and donor.

In a recent study comparing immune reconstitution after alio-HCT from MUDs with PTCy-haplo, the recovery of CD4+ and CD8+ naïve T cells was generally slow in PTCy-haplo during the first year. The pace of recovery of central memory T cells, effector memory T cells, and effector T cells was comparable (110).

In another study, the regulatory T cell-sparing effect derived from PTCy appeared in PTCy-haplo and allo-HCT from an HLA-matched unrelated donor (MUD) with PTCy (111). Early immune reconstitution by month 6 was similar in patients receiving PTCy regardless of donor type. However, the absolute numbers of helper T cell subsets, such as naïve and memory subsets stagnate between months 6 and 12 in allo-HCT from MUDs with PTCy.

A retrospective study of allo-HCT with PTCy reported a lower relapse incidence with PTCy-haplo when compared with allo-HCT from MSD (112). Another study also showed a higher trend in the cumulative incidence of relapse at 1 year in HLA-matched allo-HCT with PTCy than in PTCy-haplo (24% vs. 10%, p = 0.051) (113). Further investigation is required to determine whether these differences in post-transplant immune reconstitution have an impact on the effect of GVT.

## 12 New strategies for enhancing the GVT effect in PTCy-based allo-HCT

The MD Anderson Cancer Center reported the results of a phase I/II trial in which membrane-bound IL-21 ex vivo–expanded donor-derived NK cells were infused to decrease relapse in patients with myeloid malignancies in the early phase after PTCy-haplo (114). NK cells were expanded through a procedure using K562 feeder cells expressing membrane-bound IL21 and 4-1BBL (FC21). Three doses of donor FC21-NK cells at 1 × 10^5^–1 × 10^8^ cells/kg/dose were infused on days −2, +7, and +28. The 2-year relapse rate was 4.0% in the patients and 38% in the controls (P=.014), suggesting that adding donor-derived stimulated and expanded NK cells may effectively and optimally induce the GVT effect.

Clinical trials of novel cellular therapies based on allo-HCT with PTCy-haplo are summarized in **Table 2**.

**Table 2:** New strategies for cellular therapy in post-transplant cyclophosphamide-based transplantation.

Trial ID.	Phase	Title of trial	Intervention	Locations
NCT04836390	P2	Donor-Derived Ex-Vivo Expanded Natural Killer Cell Infusions in Children and Young Adults With High Risk Acute Myeloid Leukemia Receiving Myeloablative HLA-Haploidentical Hematopoietic Cell Transplant	Drug: donor-derived ex-vivo expanded NK cell infusions on days -1, 7 and 42.	Phoenix Children’s Hospital, United States, Children’s Hospital Los Angeles, United States, Children’s Hospital, United States, so on
NCT03524235	P1	Haploidentical Stem Cell Transplant With Prophylactic Natural Killer DLI for Lymphoma, Multiple Myeloma, and CLL	CD56-enriched donor lymphocyte infusion on day 8.	Cedars Sinai Medical Center Los Angeles, California, United States
NCT03533816	P1	Expanded/Activated Gamma Delta T-cell Infusion Following Hematopoietic Stem Cell Transplantation and Post-transplant Cyclophosphamide	The Alpha Beta (α/β) T-cell Depletion System utilizes the CliniMACS instrument to yield a gamma delta (γδ) enriched cell therapy product.	University of Kansas Cancer Center Westwood, Kansas, United States
NCT04687982	NA	Feasibility and Efficacy of Modified Donor Lymphocytes Infusion (CD45RA Negative Selected) After Haploidentical Transplantation With Post-transplantation Cyclophosphamide in Patients With Hematological Malignancies (ONC-2016-002).	CD45RA-depleted haplo-DLIs	Istituto Clinico Humanitas Rozzano, MI, Italy
NCT05250362	P1, 2	Ex Vivo Expanded NK Cells Infusion Decrease Relapse Post Hematopoietic Stem Cell Transplantation	Expanded NK cells ex vivo with a non-feeder cell IV on days 7 and 28	West China Hospital of Sichuan University, China
NCT01904136	P1, 2	Natural Killer Cells Before and After Donor Stem Cell Transplant in Treating Patients With Acute Myeloid Leukemia, Myelodysplastic Syndrome, or Chronic Myelogenous Leukemia	Expanded Natural Killer Cell Therapy on days 7 and 28-90.	M D Anderson Cancer Center, United States
NCT02782546	P2	Cytokine Induced Memory-like NK Cell Adoptive Therapy After Haploidentical Donor Hematopoietic Cell Transplantation	Cytokine Induced Memory-like NK Cell infusion on day 7.	Washington University School of Medicine, United States

PTCy, post-transplant cyclophosphamide; NK, natural kiiler; TCR, t-cell receptor; iv, intravenous injection.

## 13 Conclusion

**Figure 1:**
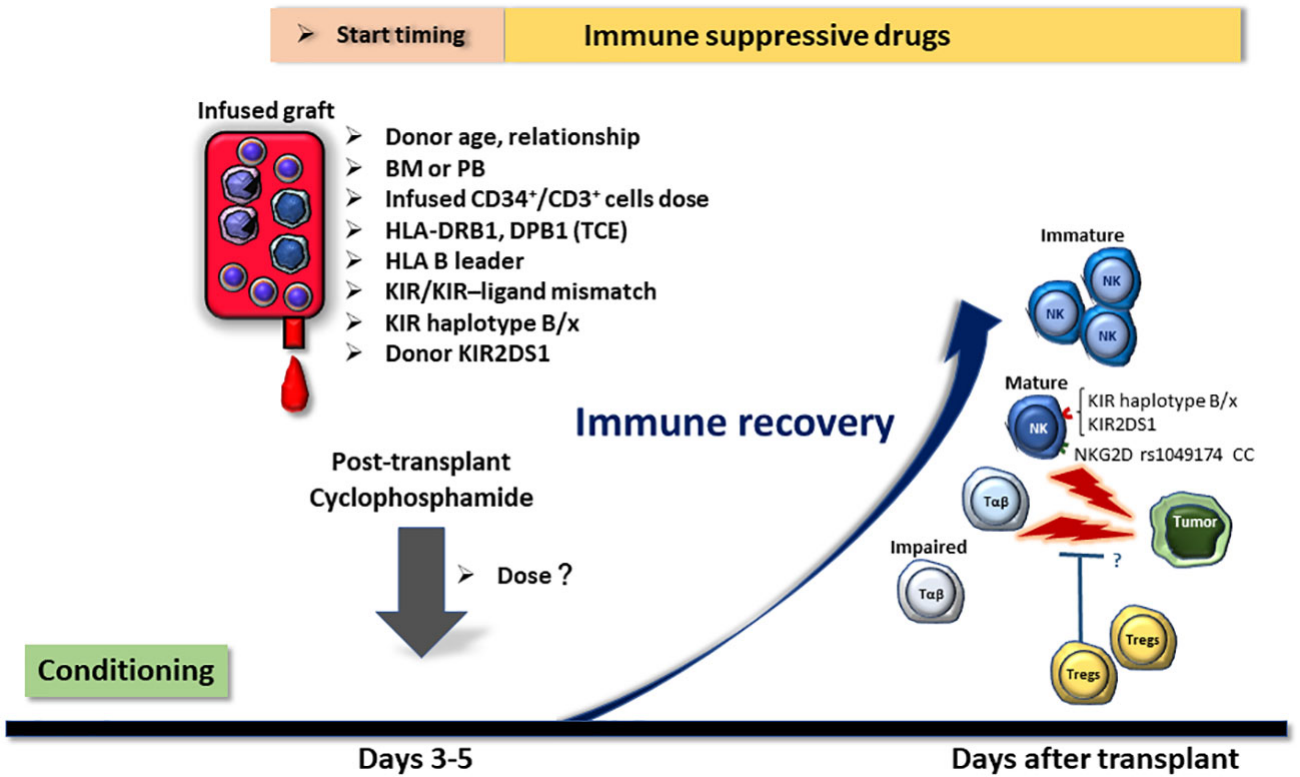
Potential factors affecting the relapse rate of post-transplant cyclophosphamide (PTCy)-based HLA-haploidentical transplantation. Factors that may influence the relapse rate of HLA-haploidentical transplantation with PTCy are displayed in the figure. Many of these variables can be selected and adjusted in actual clinical practice. Hence, it is important to establish algorithms that allow for optimal donor selection, cellular dose adjustment, and GVHD prophylaxis regimen selection without increasing the incidence of graft-versus-host disease or non-relapse mortality. BM, bone marrow; PB, peripheral blood stem cells; TCE, T-cell epitope; KIR, killer-cell immunoglobulin-like receptor.

Given the mechanism of PTCy, it may reduce the GVT effect to some extent, but there is currently no evidence that this occurs at a clinically relevant level. Various factors, including donor characteristics, type of stem cell source, count and/or type of infused cells, donor and patient HLA types, and NK KIR matching, are assumed to synergistically and/or additively contribute to the GVT effect (**Figure 1**). However, it should be noted that many of the reported factors are inconsistent across investigations; therefore, the effect sizes and importance of these factors remain unclear. Additionally, HLA-haploidentical and -matched allo-HCT may differ regarding the key factors that contribute to relapse, and further research is required.

The present review summarized the factors that influence relapse incidence. There is always a risk that inducing the GVT effect will exacerbate GVHD. Better strategies must be established to reduce the incidence of relapse without increasing the incidence of GVHD and NRM. This may lead to improved outcomes and should be investigated clinically in the setting of PTCy-based allo-HCT.

## Author contributions

HN: Conceptualization, Data curation, Funding acquisition, Investigation, Visualization, Writing – original draft, Writing – review & editing, Formal analysis.
